# Improving Decision Making for Massive Transfusions in a Resource Poor Setting: A Preliminary Study in Kenya

**DOI:** 10.1371/journal.pone.0127987

**Published:** 2015-05-28

**Authors:** Elisabeth D. Riviello, Stephen Letchford, Earl Francis Cook, Aaron B. Waxman, Thomas Gaziano

**Affiliations:** 1 Department of Medicine, Division of Pulmonary and Critical Care Medicine, Brigham and Women’s Hospital, Boston, Massachusetts, United States of America; 2 Africa Inland Church Kijabe Hospital, Kijabe, Kenya; 3 Harvard School of Public Health, Boston, Massachusetts, United States of America; 4 Department of Medicine, Division of Cardiovascular Medicine, Brigham and Women's Hospital, Boston, Massachusetts, United States of America; University Hospital Oldenburg, GERMANY

## Abstract

**Background:**

The reality of finite resources has a real-world impact on a patient’s ability to receive life-saving care in resource-poor settings. Blood for transfusion is an example of a scarce resource. Very few studies have looked at predictors of survival in patients requiring massive transfusion. We used data from a rural hospital in Kenya to develop a prediction model of survival among patients receiving massive transfusion.

**Methods:**

Patients who received five or more units of whole blood within 48 hours between 2004 and 2010 were identified from a blood registry in a rural hospital in Kenya. Presenting characteristics and in-hospital survival were collected from charts. Using stepwise selection, a logistic model was developed to predict who would survive with massive transfusion versus those who would die despite transfusion. An ROC curve was created from this model to quantify its predictive power.

**Results:**

Ninety-five patients with data available met inclusion criteria, and 74% survived to discharge. The number of units transfused was not a predictor of mortality, and no threshold for futility could be identified. Preliminary results suggest that initial blood pressure, lack of comorbidities, and indication for transfusion are the most important predictors of survival. The ROC curve derived from our model demonstrates an area under the curve (AUC) equal to 0.757, with optimism of 0.023 based on a bootstrap validation.

**Conclusions:**

This study provides a framework for making prioritization decisions for the use of whole blood in the setting of massive bleeding. Our analysis demonstrated an overall survival rate for patients receiving massive transfusion that was higher than clinical perception. Our analysis also produced a preliminary model to predict survival in patients with massive bleeding. Prediction analyses can contribute to more efficient prioritization decisions; these decisions must also include other considerations such as equity, acceptability, affordability and sustainability.

## Background

All health care occurs in the setting of finite resources, but this reality is far more conspicuous in resource poor settings. In these settings, it is apparent that using resources for futile, expensive, or ineffective treatment for one patient denies resources to other patients. Providers make decisions about how to distribute scarce resources every day, generally without data on previous outcomes and costs.

The World Health Organization (WHO) estimates that there is a worldwide shortfall of 40 million units of blood per year, primarily in developing countries.[1] Blood products are a particularly valuable resource in settings where functional national blood banks do not exist, and transfusions are based on the donations of family, friends, and hospital staff. This is the case in 75–80% of transfusions in sub-Saharan Africa.[2] Massive blood transfusion for one patient may require depleting a very limited reserve of blood products or whole blood.

The Africa Inland Church (AIC) Kijabe Hospital in Kenya is a 265-bed faith-based hospital in rural Kenya with more than 11,000 admissions, 9,000 operations, and 2000 deliveries per year. Kenya’s national blood bank was an intermittent source of blood for AIC Kijabe Hospital during the study period. In the absence of blood from the national blood bank, AIC Kijabe Hospital collected whole blood from patient family members, hospital staff, staff of a nearby boarding school, and other local community members. The hospital blood bank performed ABO and Rhesus blood-typing, as well as cross-matching, but did not screen for other alloantibodies. This study was initially prompted by a patient with a ruptured abdominal aortic aneurysm (AAA) who received eleven units of whole blood prior to dying in the hospital. The clinical staff questioned whether it was possible, in a low-resource setting, to predict which patients with indication for massive transfusion were unlikely to survive, thereby preserving resources for other patients. The manager of the blood bank estimated that fewer than half of patients receiving large transfusions survived, and recommended creating a decision-making mechanism to ensure responsible use of scarce blood.

Very few studies have looked at predictors of survival in patients requiring massive transfusion, and none has assessed the implications (in terms of health and economic outcomes) of using different criteria for deciding who will receive a massive transfusion. Relevant papers in resource-challenged settings include a study on predicting mortality for all hospitalized patients in two hospitals in Tanzania,[3] a study assessing risk factors for perioperative blood loss in prostatectomies in a Kenyan hospital,[4] and two studies designed to predict whether a patient will need massive transfusion in a combat setting.[5, 6]

Making better decisions about how to allocate the scarce resource of blood requires a better understanding of 1) the potential benefits (who is likely to survive if given blood versus those who will die whether they receive blood or not), as well as 2) the costs (direct costs, opportunity costs, and morbidity related to transfusion itself.) This study attempts to answer the first question of potential benefit by developing a model to predict who will survive with massive transfusion based on presenting characteristics.

## Methods

### Variable Selection

A few studies have looked at predictors of death after massive blood transfusion, largely focused on trauma patients, all in the developed world, and all using blood products (packed red blood cells, fresh frozen plasma, and platelets) rather than whole blood. Nonetheless, we used the available studies to guide our methods. We reviewed the relevant literature to determine potential predictor variables for our univariate and multivariate analyses. Predictive characteristics varied substantially between studies of trauma patients but included: older age, number of units of packed red blood cells transfused, hypotension on admission, arterial base deficit, Glasgow Coma Scale (GCS) < = 8, injury severity score> = 24, thromboplastin time <50%, elevated partial thromboplastin time (PTT), elevated international normalized ratio of prothrombin time (INR), head injury, intra-operative use of inotropes, aortic clamping, and intra-operative time with systolic blood pressure less than 90 mmHg.[7–15] Results are summarized in Table 1. While populations examined and definitions of massive transfusion varied widely, the table demonstrates a lack of consistent predictors. However, the trauma studies universally conclude that a threshold for futility could not be defined, based on relatively high survival rates and the fact that the volume of blood transfused was not a consistent independent predictor of death.[7, 8, 10–13, 16, 17]

**Table 1:** Studies with predictors of mortality in patients receiving massive transfusions.

Study	Inclusion criteria	Number of patients	Predictors of mortality	Overall survival
Campos 2007^a^	• > = 8 units pRBC within 24 hour period	288	• Age • Number of units of pRBC in 24 hrs • Non-trauma cause of bleeding	52%
Chojkier 1986^b^	• Upper gastrointestinal bleed • HCT decrease> = 6% • Unstable vital signs• > = 2 units pRBC	100	• Inpatient at time of bleed • Number of life-threatening co-morbidities • Number of units of blood products	65%
Como 2004^c^	• Trauma • >10 units pRBC	147	• Number of units of pRBC	61%
Criddle 2005^d^	• Trauma • > = 50 units blood products in first day post-injury	46	• ISS • Arterial base deficit	63%
Huber-Wagner 2007^e^	• Trauma • > = 10 units of Prbc	1062	• Age >55 yrs • GCS< = 8 • Number of units of pRBC > = 20 • Thromboplastin time<50% • ISS> = 24	56.9%
Mahambrey 2009^f^	• > = 10 units pRBC within 24 hours of admission	260	• Age • ISS • SOFA score • Nadir hemoglobin at <48 hrs • Total units of blood transfused <48 hours	41.5%
Mitra 2007^g^	• Trauma • > = 5 units pRBC within 4 hrs	119	• ISS • PTT • Head injury	72.3%
Turan 2013^h^	• Non-cardiac surgery • > = 5 units pRBC	5,143	• Age • ASA class • Emergency case • Surgical types • Coma>24h before surgery • Sepsis • INR • Number of intraoperative transfusions • Post-operative transfusion requirement	78.5%
Vaslef 2002^i^	• Trauma • >50 units blood products in 24h	44	• Base deficit > 12 mmol/L	43%
Velmahos 1998^j^	• Trauma • > = 20 u pRBC or whole blood pre-operatively and intra-operatively	141	• Need for aortic clamping • Intraoperative inotropes • SBP< = 90 mmHg intra-operatively	30.5%
Wudel 1991^k^	• Blunt trauma • > = 20 u pRBC	92	• Shock on admission • Closed head injury • Age	52%

pRBC = packed red blood cells. ISS = injury severity score. GCS = Glasgow coma scale. SOFA = sequential organ failure assessment. PTT = partial thromboplastin time. ASA = American Society of Anesthesiologists. INR = international normalized ratio (of prothrombin time). SBP = systolic blood pressure.

^a^Campos A, Munoz M, Garcia-Erce JA, Ramirez G. [Incidence and mortality of massive transfusion in a university hospital: study of the period 2001–2005]. Med Clin (Barc). 2007;129(10):366–71.

^b^Chojkier M, Laine L, Conn HO, Lerner E. Predictors of outcome in massive upper gastrointestinal hemorrhage. J Clin Gastroenterol. 1986;8(1):16–22.

^c^Como JJ, Dutton RP, Scalea TM, Edelman BB, Hess JR. Blood transfusion rates in the care of acute trauma. Transfusion. 2004;44(6):809–13.

^d^ Criddle LM, Eldredge DH, Walker J. Variables predicting trauma patient survival following massive transfusion. J Emerg Nurs. 2005;31(3):236–42; quiz 320.

^e^ Huber-Wagner S, Qvick M, Mussack T, Euler E, Kay MV, Mutschler W, et al. Massive blood transfusion and outcome in 1062 polytrauma patients: a prospective study based on the Trauma Registry of the German Trauma Society. Vox Sang. 2007;92(1):69–78.

^f^Mahambrey TD, Fowler RA, Pinto R, Smith TS, Callum JL, Pisani NS, et al. Early massive transfusion in trauma patients: Canadian single-centre retrospective cohort study. Can J Anaesth. 2009;56(10):740–50.

^g^Mitra B, Mori A, Cameron PA, Fitzgerald M, Street A, Bailey M. Massive blood transfusion and trauma resuscitation. Injury. 2007;38(9):1023–9.

^h^Turan A, Yang D, Bonilla A, Shiba A, Sessler DI, Saager L, et al. Morbidity and mortality after massive transfusion in patients undergoing non-cardiac surgery. Can J Anaesth. 2013;60(8):761–70.

^i^Vaslef SN, Knudsen NW, Neligan PJ, Sebastian MW. Massive transfusion exceeding 50 units of blood products in trauma patients. J Trauma. 2002;53(2):291–5; discussion 5–6.

^j^Velmahos GC, Chan L, Chan M, Tatevossian R, Cornwell EE, 3rd, Asensio JA, et al. Is there a limit to massive blood transfusion after severe trauma? Arch Surg. 1998;133(9):947–52.

^k^Wudel JH, Morris JA, Jr., Yates K, Wilson A, Bass SM. Massive transfusion: outcome in blunt trauma patients. J Trauma. 1991;31(1):1–7.

### Data collection

Patients who received five or more units of whole blood within 48 hours between April 2004 and April 2010 were identified from the blood registry of AIC Kijabe Hospital. The definition of “massive transfusion” is highly variable between studies; we used the above threshold in order to maximize inclusivity and in recognition of the fact that five units of blood in 48 hours is an unusually large transfusion in this setting. Our definition represents the historic definition of > = 50% of total blood volume and is consistent with the majority of prior studies outlined in Table 1, with only one study having a threshold less than five units. The number of units transfused in 48 hours and during entire hospitalization for each patient was also collected from the registry. In-hospital survival was collected from patient charts. The following variables were also collected from charts: gender, age, home district and village, admission date, discharge or death date, admission co-morbidities, reason for admission and primary diagnosis, indication for transfusion, initial vital signs, vital signs just prior to first transfusion, GCS, first recorded hemoglobin, last hemoglobin prior to transfusion, any INR, PTT or platelets recorded prior to transfusion, whether the patient entered the intensive care unit (ICU), blood group, operations performed with dates, whether the patient presented from home or was transferred from another facility, and whether the admission was a planned admission for surgery. Indication for transfusion was categorized as obstetric complications, non-urgent surgery, urgent surgery, trauma and other.

Institutional Review Board approval was obtained from the AIC Kijabe Hospital Ethics Committee in Kijabe, Kenya and the Partners Human Research Committee in Boston, U.S.A. Both committees waived the need for written informed consent from participants because it was not feasible to contact patients or next of kin for this retrospective study and because the potential risk to participants was deemed to be minimal. Patient records accessed for the study were maintained in a locked secure office at AIC Kijabe Hospital and were only accessed by study investigators and staff. Records were returned to the hospital archives immediately after data had been extracted. All patient information was anonymised and de-identified prior to analysis.

### Statistical methods

Analyses were conducted using SAS software version 9.3. We analyzed whether the number of units transfused in the initial two days, or the total number of units transfused during hospitalization, predicted in-hospital mortality using a Pearson chi-square statistic. We then analyzed whether any presenting patient characteristics were predictors of mortality. A Pearson chi-square statistic was calculated for each categorical variable thought to be a possible predictor of in-hospital mortality, and a T-test for each continuous variable thought to be a possible predictor of mortality. We also categorized all continuous variables and calculated a Pearson chi-square statistic for these categorical variables. Using forward selection, a multivariable logistic regression model was developed to predict who would benefit (i.e., in-hospital survival) from massive transfusion versus those who would die despite transfusion. We chose the most predictive variables from the univariate analyses based on p values, and added them into the multivariate model in a stepwise fashion, conforming to the rule of thumb allowing only one predictive variable for every 5–10 outcome events to avoid overfitting the model. An ROC curve was created from this model.

## Results

Ninety-eight patients met inclusion criteria. Three charts were missing after extensive searching, so the dataset consisted of the remaining 95 observations. Seventy-four percent survived to discharge. Indications for transfusion were: obstetric complications (18%), non-urgent surgery (33%), urgent surgery (11%), trauma (18%), and other (20%). “Other” diagnoses included gastrointestinal bleeds, hematologic disorders, and uterine fibroids. The mean age was 46, with a range from 14 to 88 years; 41 participants (43.2%) were male.

**Fig 1:**
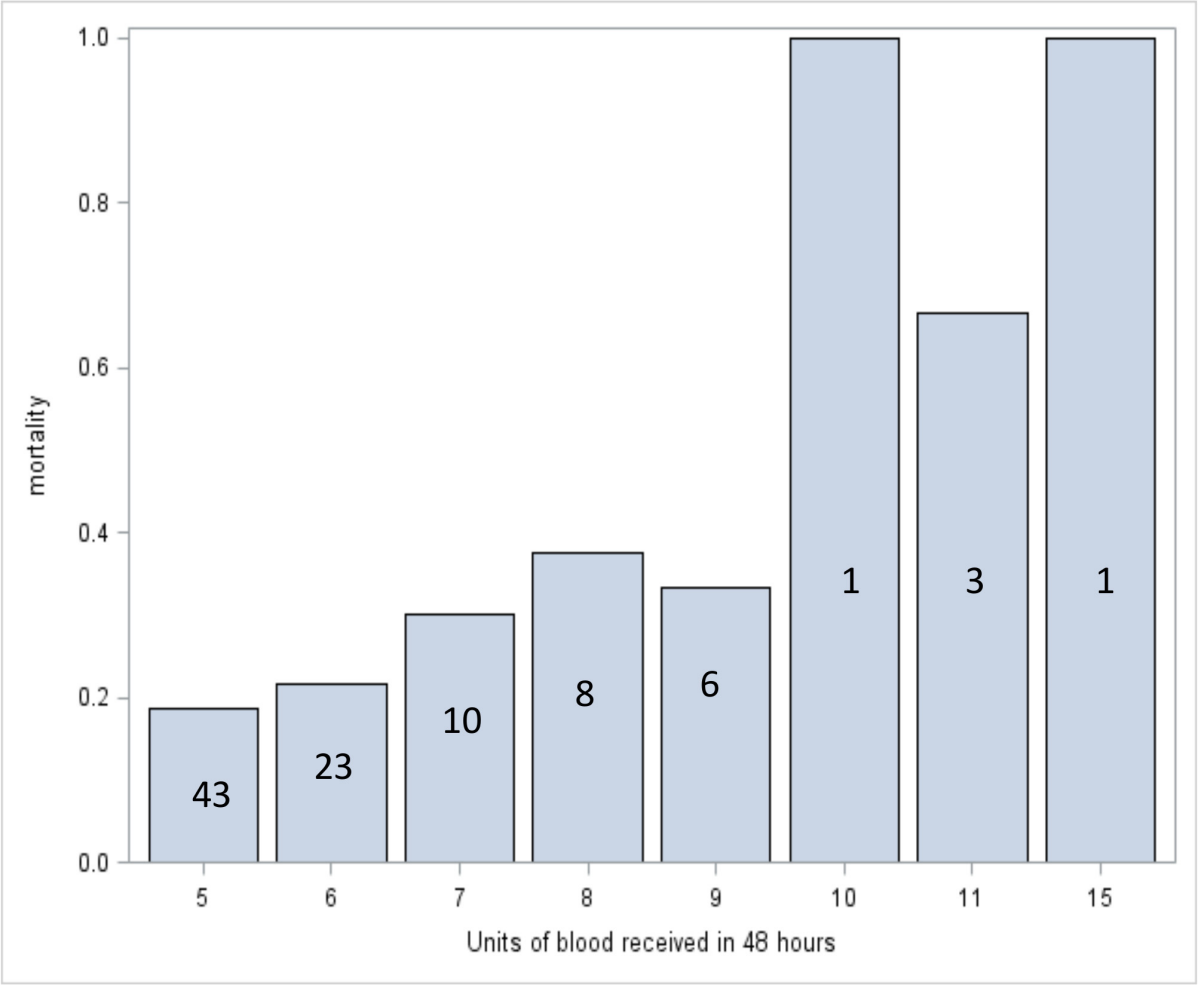
Mortality rates stratified by number of units of blood received within a 48 hour period. Numbers superimposed on the bars indicate the number of patients represented in each bar. P = 0.166.

**Fig 2:**
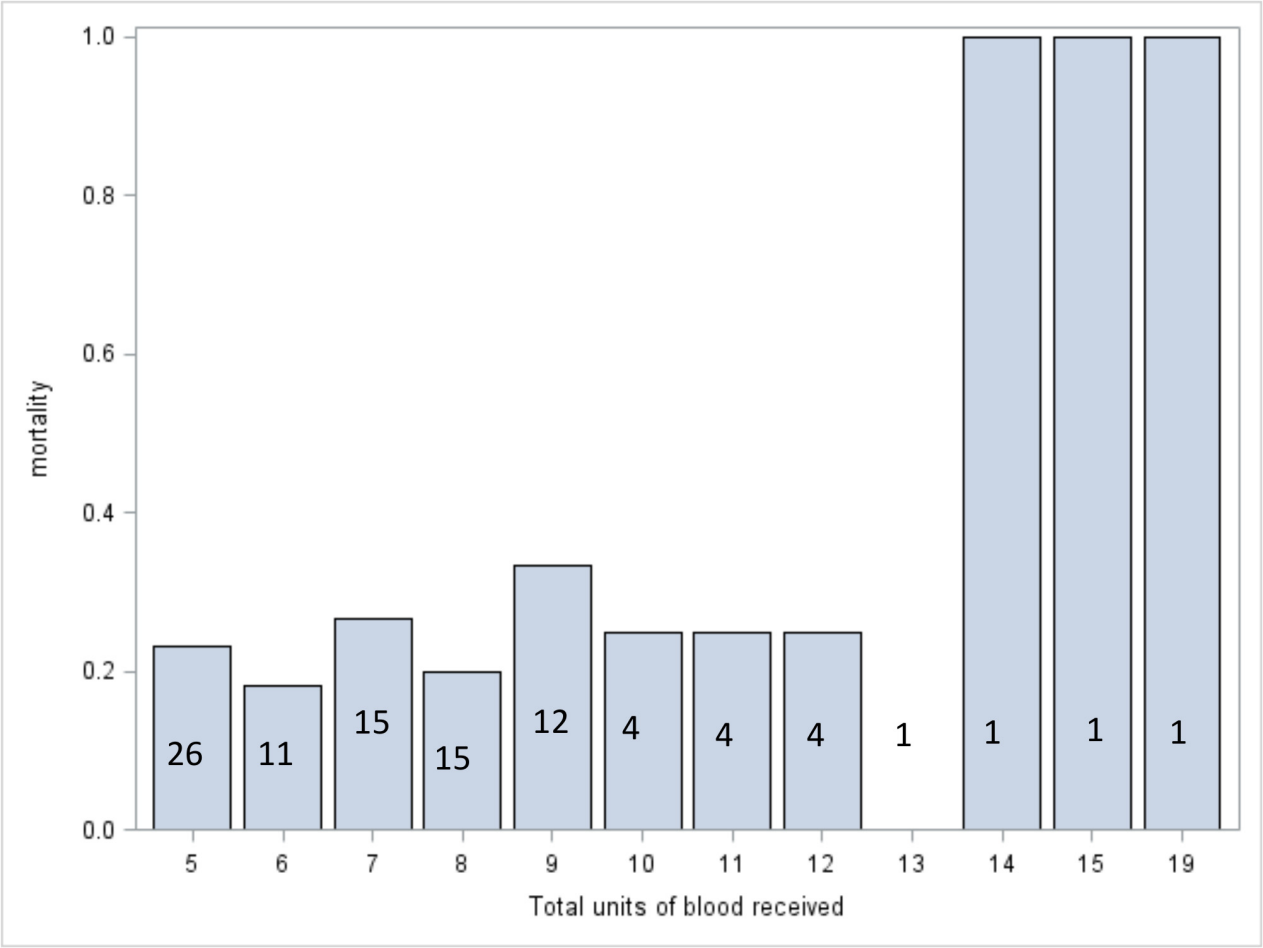
Mortality rates stratified by total number of units of blood received during hospitalization. Numbers superimposed on the bars indicate the number of patients represented in each bar. P = 0.540.

Neither the number of units transfused in 48 hours nor the total number of units transfused during the hospitalization was a statistically significant predictor of mortality. See Figs 1 and 2 for mortality stratified by number of units transfused. While mortality is higher in the highest transfusion categories, numbers in these strata are small.

A Pearson chi-square statistic was calculated for all variables hypothesized to predict death (Table 2). We analyzed blood pressure and heart rate as potential predictors using values at the time of hospital presentation and values just prior to transfusion. The other vital signs were frequently missing at the time of hospital presentation, so we analyzed these as values recorded at any time before transfusion. We analyzed indication for transfusion in two ways: as a categorical variable with the five categories noted above, and using trauma and obstetric patients as a combined category compared to all other patients. We performed this analysis because both trauma and obstetric patients tend to be young and otherwise healthy; we hypothesized that these indications for transfusion might be similarly protective. The following were found to be the best predictors of death:

- low blood pressure on presentation, defined as systolic blood pressure (SBP)<90 or mean arterial pressure (MAP)<60 (p = 0.0155),
- presence of any known comorbidities on presentation (p = 0.0417), and
- indication for transfusion being something other than trauma or obstetrics (ie, urgent surgery, non-urgent surgery, or other) (p = 0.0550).

Initial hypotension was present in 14% of patients; comorbidities in 39%; and indication for transfusion other than trauma or obstetrics in 65%.

**Table 2:** Univariate analyses of potential predictor variables.

Potential predictor variables	P values
Initial hypotension (SBP<90 or MAP<60)	0.0155
Presence of comorbidities	0.0417
Indication for transfusion other than obstetric emergency or trauma	0.055
Transferred from an outside facility	0.125
Male gender	0.167
Low hemoglobin (<7 g/dL)	0.196
Age > 65 years old	0.274
Known abdominal aortic aneurysm	0.299
Low oxygen saturation (<90%)	0.319
Unplanned admission	0.335
Abnormal temperature (<36 or >38 C)	0.354
Indication for transfusion (five categories)	0.383
Tachycardia (HR>100)	0.591
Initial tachycardia (HR>100)	0.601
Abnormal platelet or coagulation labs	0.603
Glasgow coma scale (GCS) <8	0.714
High respiratory rate (RR>24)	0.768
Hypotension (SBP<90 or MAP<60)	0.852
Distance from hospital	0.960

P values for chi square analyses with mortality as outcome. Initial hypotension is defined as systolic blood pressure (SBP)<90 or mean arterial pressure (MAP)<60 at the time of hospital presentation. Presence of comorbidities refers to any past medical history listed in the patient’s chart on hospital presentation. Low hemoglobin is a hemoglobin < 7 g/dL at any time before transfusion. Low oxygen saturation is a saturation <90% at any time before transfusion. Abnormal temperature is a temperature >38 or <36 degrees Celsius at any time before transfusion. Indication for transfusion (five categories) looked at whether any individual reason for transfusion (obstetric emergency, trauma, planned surgery, unplanned surgery, or other) were associated with mortality. Tachycardia refers to a heart rate (HR)>100 beats per minute at time of presentation or just before transfusion. Initial tachycardia refers to HR>100 beats per minute at time of hospital presentation. Abnormal platelet or coagulation labs refers to international normalized ratio of prothrombin time (INR)> = 1.5, partial thromboplastin time (PTT)> = 45 seconds or platelets<150 K/uL. Hypotension refers to SBP<90 or MAP<60 on presentation or just prior to transfusion. Distance from hospital is a categorical variable with categories of approximately equal size: 3–35, 36–100, 101–200, and >200 kilometers from the hospital.

**Fig 3:**
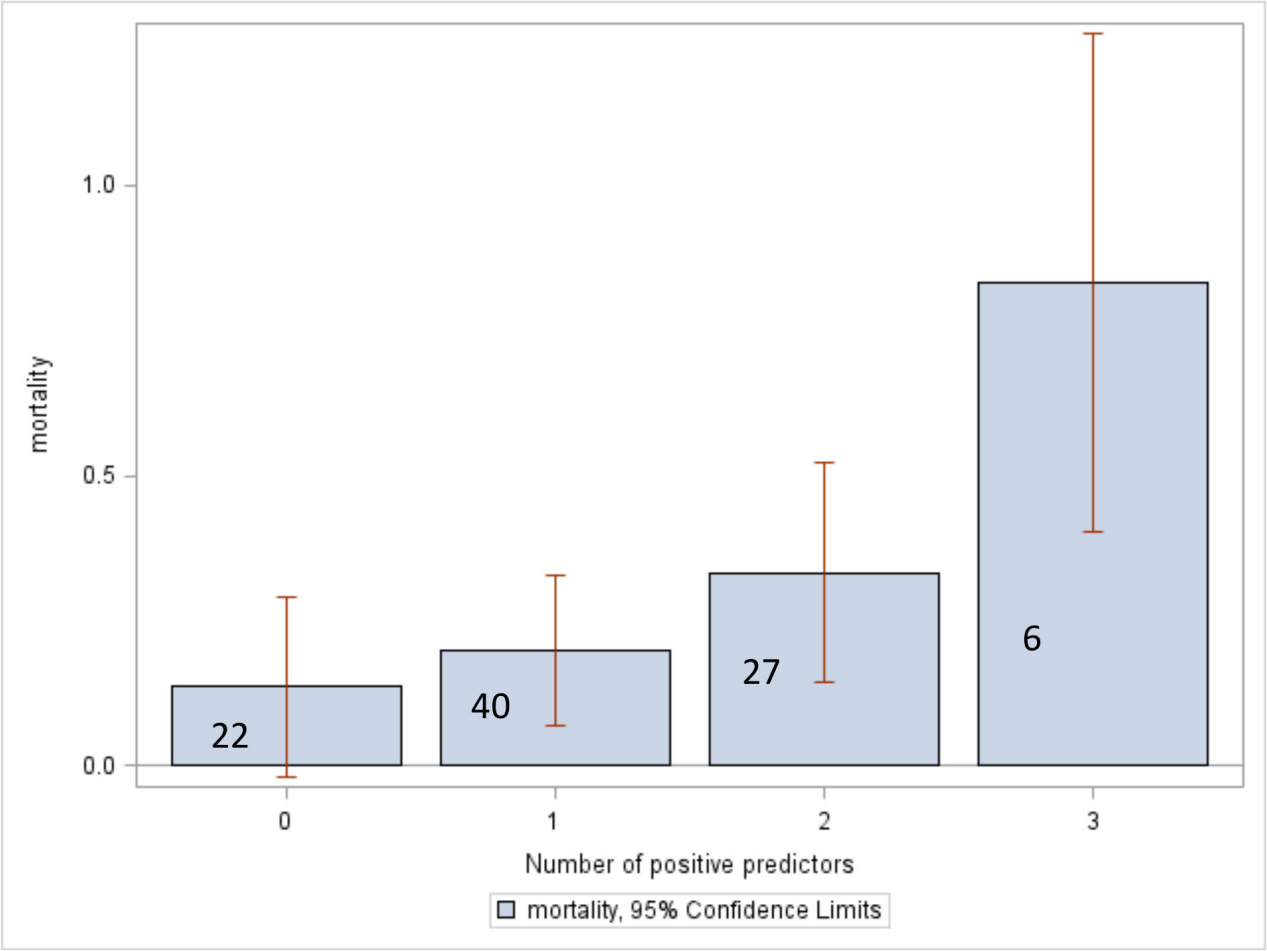
Mortality stratified by the number of risk factors present. The risk factors are: hypotension at presentation, presence of co-morbidities at presentation, and non-trauma or obstetric indications for transfusion. Numbers superimposed on the bars indicate the number of patients in each risk factor category. Red lines indicate confidence intervals. P = 0.0039.

Fig 3 demonstrates mortality rates for patients stratified by the number of the top three risk factors present. The next strongest predictors of in-hospital death (not used in the logistic model) were: whether a patient had been transferred from another facility (p = 0.125), male gender (p = 0.167), hemoglobin prior to transfusion less than 7 g/dL (p = 0.196), and age greater than 65 years (p = 0.274) (Table 2).

**Fig 4:**
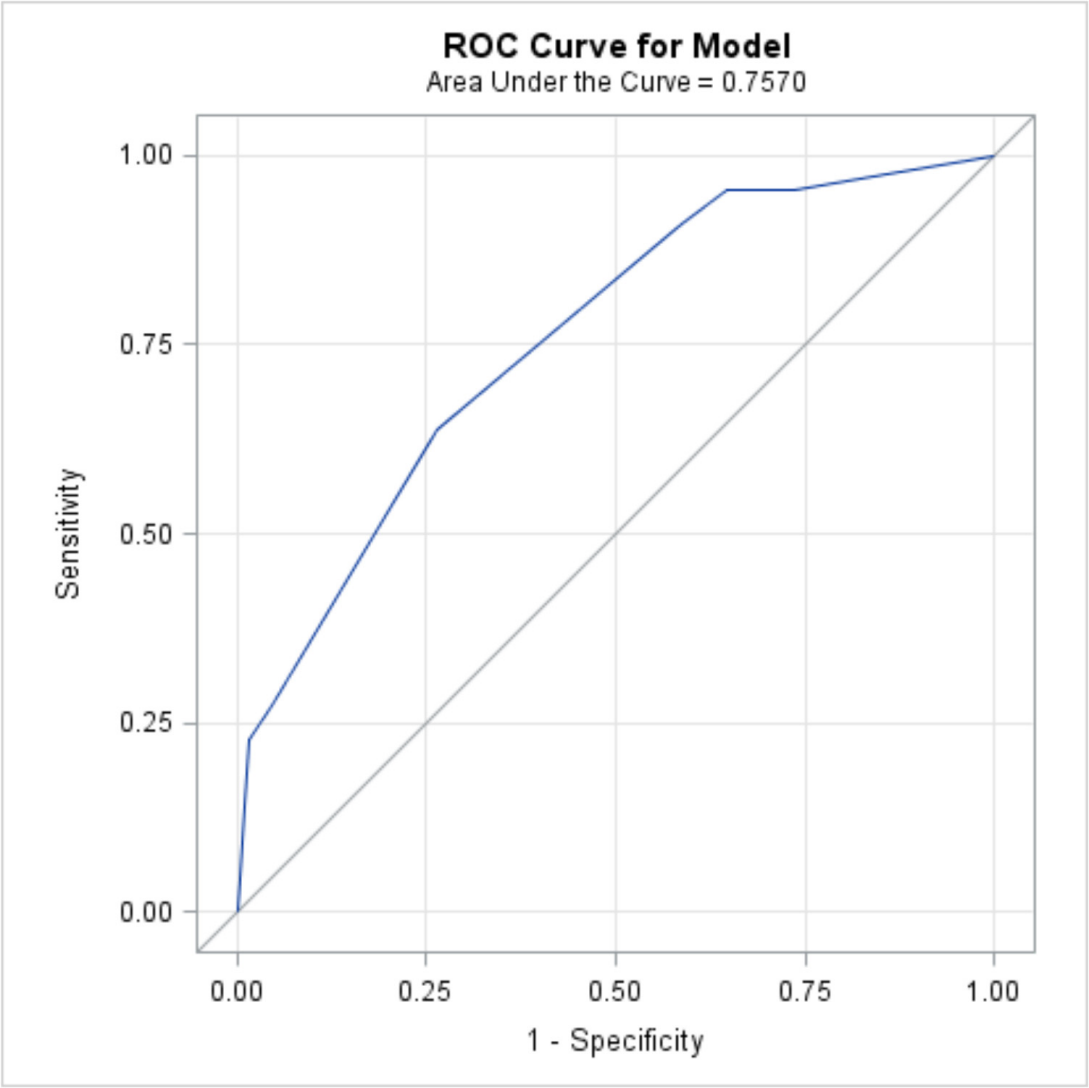
Receiver operating characteristic (ROC) curve for the logistic regression model predicting mortality based on initial hypotension, co-morbidities, and indication for transfusion. The area under the curve is 0.757.

Using the three most significant variables, multivariable logistic regression was performed. The odds ratio estimates for the variables in the model were: 5.225 for hypotension, 2.115 for comorbidities, and 6.662 for an indication other than obstetrics or trauma (Table 3). An ROC curve was created from the three-variable model, with area under the curve (AUC) of 0.757 (Fig 4). Bootstrap validation estimates that this is optimistic by 0.023, so we estimate that using this model on a separate validation data set would give an AUC of 0.734.

**Table 3:** Results of the final logistic model for predicting mortality.

Predictor variables	Odds ratio estimates	95% Wald Confidence Limits
Initial hypotension (SBP<90 or MAP<60)	5.225	1.311, 20.818
Presence of comorbidities	2.115	0.709, 6.306
Indication for transfusion other than obstetric emergency or trauma	6.662	1.297, 34.218

## Discussion

Our retrospective analysis of a small population is the first study of mortality prediction in massive transfusion in a resource poor setting. Neither the number of units transfused over 2 days nor the total units transfused was significantly related to in-hospital survival; in neither case was it possible to set a limit of futility above which people should not be transfused. Our study is different from the previous developed-world studies in that it takes place in a context of far fewer overall medical resources, analyzes transfusion of whole blood rather than blood products, includes only limited ABO blood typing and matching, and uses a threshold for “massive transfusion” that is on the low end of units transfused and the high end of transfusion period. Nonetheless, our results are consistent with the developed world trauma data that found it impossible to define a point of futility due to high survival even with very large transfusions, as well as lack of predictive power of the total number of units transfused.[7, 8, 10–13, 16] It should be noted that we were not able to perform a time-varying co-variate analysis with our data, so it is possible that the lack of association between total units transfused and mortality reflects the fact that patients who survive longer are more likely to receive additional transfusions.

We demonstrated that initial hypotension and indication for transfusion are predictors of in-hospital mortality in one hospital in Kenya; presence of comorbidities is also likely a predictor though its confidence interval includes one in the multivariate model. None of these is surprising; however it is noteworthy that some other factors that might be expected to impact survival, such as age, home distance from the hospital, or initial tachycardia, were not as predictive. The model had modest predictive power, but provides a basis for testing its validity in another population.

This preliminary study has several strengths. It is the first of its kind in a resource-limited setting. In addition, we are fortunate to work in a setting where blood is available acutely; this means that we did not encounter the potential bias of excluding patients who died due to lack of immediate blood availability. The finding that prognosis for patients requiring massive transfusion was better than predicted by practitioners, and could not be defined by number of units transfused, is consistent with developed world studies of transfusion and is important for guiding decision-making in this real-world setting. Our model itself, while only modestly predictive, is nonetheless a useful starting place, and we hope will be tested in other resource-challenged settings.

The study has a number of limitations. First, the sample size is small, resulting in an inability to validate our model. We were able to estimate the optimism of our model using bootstrap validation, but an important next step is to validate the model in another population. Second, the registry and charts from which data were obtained were not complete. Pages of the registry were occasionally missing or ripped and were at times difficult to interpret. We checked information in as many sources as possible, and each observation’s data sheet was reviewed by at least two people, but inaccuracies in data keeping may have affected results in this retrospective chart review. Third, the model has only modest predictive power, with an area under the curve of 0.757 and optimism of 0.023, which may be in part due to the small sample size. Fourth, the logistic model was created using stepwise selection, implying that its fit to the data is overly optimistic. Finally, our definition of massive transfusion as > = 5 units of whole blood within 48 hours is such that our study contains less-severely-ill patients than most studies in the developed world. While we are aware that this may increase the heterogeneity of our population and decrease its comparability to developed world studies, we nonetheless think this population is relevant to other resource-poor settings in which five units of blood over 48 hours is an unusually high expenditure of resources.

Prognostication scores are largely used only for research, to control for disease severity across study arms. While they could be used for resource allocation decisions, they rarely are in practice. Cost effectiveness models can provide more information on which to base resource allocation decisions since they incorporate both costs and benefits of various choices; some of the best examples of these models are in HIV care.[18] Further studies in other populations receiving massive transfusions with additional cost and survival data would be helpful for informing policy makers.

Our study demonstrated that even prognostication data alone provides important information that can be used for decision-making. For example, prior to the current analysis, one experienced blood bank manager at AIC Kijabe Hospital perceived that fewer than half of patients receiving massive transfusions survived. The finding that 74% of massive transfusion patients survived provides data for more informed decisions. The local impetus for this study was the question of futility raised by a patient who received a total of eleven units of blood for a ruptured abdominal aortic aneurysm (AAA) and died; the data show that 75% of all patients who receive a total of eleven units survive. The presence of a ruptured AAA was not a predictor of mortality in our small study that included six patients with AAA requiring urgent surgery. The model points to three variables that clinicians might consider as they are looking at potential survival of patients in need of massive transfusion. Resource allocation decisions must be made, and it is valuable to add prognostication data to other considerations including equity, acceptability, affordability and sustainability. This paper is a preliminary analysis, but represents an example of what may be possible in beginning to fill the dearth of information on benefits and costs when making allocation decisions with scarce resources.

## Supporting Information

S1 Dataset Massive transfusion dataset. (XLSX)
